# Orosomucoid: a promising biomarker for the assessment of exercise-induced fatigue triggered by basic combat training

**DOI:** 10.1186/s13102-022-00490-6

**Published:** 2022-06-03

**Authors:** Yi Ruan, Ke-fa Xiang, Hui-min Zhang, Zhen Qin, Yang Sun, Jing-jing Wan, Wei Gu, Xia Liu

**Affiliations:** 1 Department of Pharmacology, School of Pharmacy, Naval Medical University (Second Military Medical University), Shanghai, 200433 China; 2 School of Traditional Chinese Medicine, Naval Medical University (Second Military Medical University), Shanghai, 200433 China

**Keywords:** Exercise-induced fatigue, Orosomucoid, Basic combat training, Biomarker

## Abstract

**Background:**

Orosomucoid (ORM) is a positive acute phase protein verified to be upregulated in various forms of exercise-induced fatigued (EIF) rodents. However, its association with EIF among human beings remained unknown. This study aimed to explore the association between serum ORM and EIF triggered by military basic combat training (BCT).

**Methods:**

The degree of EIF were measured by Borg’s Rating of Perceived Exertion Scale (Borg-RPE-Scale®) as RPE score after BCT. Fifty-three male recruits were classified into three groups according to the RPE score: (1) group 1 (slight fatigue group): RPE score after BCT < 13; (2) group 2 (moderate fatigue group): RPE score after BCT = 13 or 14; (3) group 3 (severe fatigue group): RPE score after BCT > 14. The levels of blood ORM, lactate (LAC), cortisol and C-reactive protein (CRP) were determined before and after BCT. The diagnostic value of ORM was evaluated by receiver operating characteristic (ROC) curve analysis and logistic regression.

**Results:**

After BCT, the level of LAC, CRP, and cortisol increased among all groups, but the changes had no significant between-group difference (all *p* > 0.05). The level of ORM had a specific significant increase in group 3 (*p* = 0.039), and the changes of ORM (ΔORM) had significant difference among groups (*p* = 0.033). ROC curve analysis showed that the estimated area under ROC curve for ΔORM was 0.724 (*p* = 0.009) with the recommended optimal cut-off value as 0.2565 mg/mL. Logistic analysis showed that recruits with ΔORM ≥ 0.2565 mg/mL had higher odds for suffering from severe EIF, 5.625 times (95% CI 1.542–20.523, *p* = 0.009) as large as those with ΔORM < 0.2565 mg/mL.

**Conclusion:**

ORM might be a promising biomarker of severe EIF triggered by BCT among male recruits. Its potential optimal cut-off value regarding ΔORM was recommended to be 0.2565 mg/mL.

**Supplementary Information:**

The online version contains supplementary material available at 10.1186/s13102-022-00490-6.

## Background

Exercise-induced fatigue (EIF) refers to a state in which the physiological function of the body cannot be kept at a certain level or the organism cannot maintain a predetermined exercise intensity [1].

The precise pathophysiological mechanism of EIF remains unclear now. Some evidences show that the occurrence of EIF correlates with both physical (e.g., training intensity and volume) and social-psychological factors (e.g., feeling of loneliness, resistance psychology to exercise) [2–5], which may include central and peripheral nerve-muscle functional activities [6], cardiovascular and respiratory system functions [7], energy and substance metabolism [8], internal environment disorders [9], and collapse of fatigue control chain [10]. The uncertainty of the mechanism results in the uncertainty of objective evaluation of EIF. At present, the confirmed and widely-acknowledged tool of EIF assessment is the Borg’s Rating of Perceived Exertion Scale, a widely used psycho-physical tool to semi-quantitatively assess subjective perception of EIF [11], but objective specific indicator for the evaluation of EIF is still absent.

In China, basic combat training (BCT), formed from high-intensity military training, is a stage helping a recruit become a qualified soldier [12]. Fatigue among military personnel can be triggered by various factors, among which, BCT is the potential primary one [13]. As reported by a meta-analysis of 52,597 recruits, during BCT period, EIF is one of the main causes of military training injury [14]. Therefore, assessment and management of EIF has strategic significance for maintaining combat capability [15], which starves for some specific and objective indicators or biomarkers for the objective assessment of EIF.

Orosomucoid (ORM) is a positive acute phase protein (molecular weight: 37–54 kDa) verified to modulate inflammation, immunity, mediate the energy metabolism, and maintain the function of capillary [16]. A previous study verified that in rodents suffering from exercise-induced fatigue, the level of ORM was upregulated significantly in blood, muscle and liver [17]. Also, it is verified that ORM is a potential biomarker for the diagnosis of chronic fatigue syndrome [18]. These results suggested that ORM may be a potential biomarker for the assessment of EIF triggered by BCT.

According to the previous studies, blood lactic acid (LAC) and cortisol are two typical traditional biomarkers of fatigue. LAC is the anaerobic breakdown metabolite of carbohydrate. During exercise and physical training, as the oxygen and energy exhaust, the metabolite LAC accumulates [19]. The “LAC hypothesis” for muscle fatigue, a kind of EIF, states that “accumulation of lactate or acidosis in working muscle causes inhibition of contractile processes, either directly or via metabolism, resulting in diminished exercise performance” [20]. At present, LAC, as an indicator of energy metabolism, has been regarded as a biomarker of EIF in some intensive exercises such as swimming [21, 22] and cycling [23, 24]. Cortisol is a kind of hormones maintaining the body homeostasis produced during acute or chronic physical exercise [25]. Study of Dely et al. [26] reported that the level of cortisol doubled immediately when participants exercised at 100% of their ventilatory threshold on a treadmill until volitional fatigue. Kageta et al. [27] also observed similar exercise-induced response of cortisol during a three-week chronic exercise training. Furthermore, cortisol has been reported to regulate the expression of *ORM* gene in liver cells [28].

CRP, an analogues of ORM, is also an acute-phase protein. Increased levels of CRP have been observed in fatigue of patients suffering from chronic hemodialysis [29] and rheumatoid arthritis [30]. Some studies reported that regular exercise appeared to decrease CRP level [31–33], while contrary conclusion was also reported by studies of physical activity [34, 35].

In this study, we aimed to explore the association between serum ORM and EIF triggered by BCT among male recruits undergoing Chinese BCT, so as to clarify the efficiency of ORM in the assessment of EIF. Besides, we also determined the levels of LAC, cortisol and CRP to verify whether ORM is the more useful biomarker than the classical parameters for the diagnosis of EIF triggered by BCT.

## Methods

### Participants and BCT

From the end of September to December in 2018, we followed 53 male recruits undergoing BCT at a navy basic training base in China. This navy BCT is a 12-week high-intensity military training course covering basic physical training and navy tactical training, according the requirements of the Chinese Naval Military Training Program [36]. The basic physical training covers various military endurance trainings, strength trainings, and speed trainings. The navy tactical training includes normal tactical skills and actions. The BCT was uninterrupted during 12 weeks. Daily training items were scheduled by this training base, which usually comprised several types of physical training and navy tactical training.

The inclusion criteria of participants were: (1) qualified recruits in this navy basic training base in 2018; (2) aged 18 to 25; (3) no major organic diseases or limb joint damage that may prevent participation in daily military training tasks; (4) willing to participate in this study and promising the authenticity of the subjective survey questions; and (5) signing the informed consent. The exclusion criteria of participants were: (1) having taken one or more drugs that may cause fatigue within the 2 weeks prior to BCT and (2) suffering from diseases that may cause fatigue, such as kind of acute or chronic infectious diseases (e.g., colds), fibromyalgia, depression, tristimania, etc., within the 2 weeks prior to BCT.

All the recruits were managed with unified training, life schedule, and diet supplies by the training base. According to the average diet supplies, no caffeine was involved in all the diet of recruits.

This study was approved by the Shanghai Changhai Hospital Ethnics Committee (No: 2018-048), and performed in accordance with the Declaration of Helsinki.

### Measurement of EIF and group classification

According to the definition of EIF [1] and the current methods of EIF assessment, the degree of EIF was primarily evaluated by subjective perception scale, while physical exercise capacity was also determined to further clarify the rationale of subjective perception.

Subjective perception of EIF were measured by Borg’s Rating of Perceived Exertion Scale (Borg-RPE-Scale®) as RPE score before (one day before the beginning of BCT) and after BCT (immediately when participants finished the last item of BCT physical assessment). The BCT physical assessment was performed at the last day of this 12-week BCT, which includes three typical physical training items and one typical navy tactical training item. The grade of BCT physical assessment would influence a recruit’s occupation allocation. Therefore, recruits always make their every effort to get a better grade, which often results into fatigue.

**Fig. 1:**
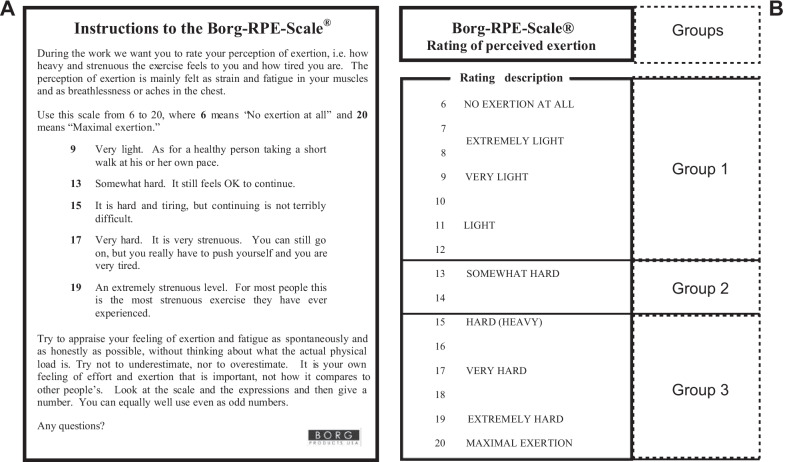
Borg-RPE-Scale® (**A**) and classification of groups (**B**)

The Borg-RPE-Scale®, widely suggested as a valid method for monitoring EIF, has 15 rates in total, from 6 (no exertion at all) to 20 (maximal exertion), with a higher rate score indicating a higher degree of perceived fatigue [37]. It is reported that the RPE score is closely related to exercise load intensity, heart rate, oxygen consumption, lactic acid, and hormones [38]. Generally, RPE < 9 indicted the feeling of very light exertion like a healthy person taking a short walk, RPE = 13 indicated the physical activity is somewhat hard for a person but to continue is no problem, and when RPE increases to 15, the person feels tiring and has difficulty to continue the activity (Fig. 1A) (Borg G. Borg’s Perceived Exertion and Pan Scales. Champaign, IL: Human Kinetics, 1998). According to the above classification of Borg-RPE-Scale® score, participants were classified into three groups according to the RPE score measured after BCT as follows: (1) group 1 (slight fatigue group): RPE score after BCT < 13; (2) group 2 (moderate fatigue group): RPE score after BCT = 13 or 14; (3) group 3 (severe fatigue group): RPE score after BCT > 14 (Fig. 1B).

Physical exercise capacity was measured by TZCS-3 Vertical Jump Height Monitor (KeDao Electronic Co. Ltd, Ningbo, China) as vertical jump height (VJH) before (one day before the beginning of BCT) and after BCT (immediately when participants finished the last test item of BCT physical assessment). VJH is a simple test to evaluate exercise capacity without extra strenuous exercise and has been indexed in the Handbook of National Physical Fitness Determination Standards in China [39]. Some studies have addressed that by indicating the capacity of lower limb neuromuscular state, VJH could also reflect the degree of EIF [40, 41]. Although there are also various other tests for the determination of exercise capacity, generally to finish those tests, an extra strenuous exercise is inevitable. Hence, in this study, to avoid the impact of Physical exercise capacity measurement on the degree of EIF, we chose VJH as the index.

### Measurement of blood ORM, LAC, cortisol and CRP

On considering the potential properties of EIF assessment or their association with ORM, we included LAC, cortisol, and CRP as indicants for comparison. The blood samples of participants were collected before (the morning before the beginning of BCT, week 0) and after BCT (immediately when participants finished the last test item of BCT physical assessment, week 12) from the cubital vein. The blood samples were collected in inert separation gel vacuum collective tubes (BD Vacutainer SST II Advance Tubes, 5 mL, #367,955, BD, UK) and immediately served in 0 ℃ circumstance. Within 20 min after blood drawing, all the collected blood samples were centrifuged at 3000 rpm for 5 min to retrieve the serum. Serum levels of ORM were determined via Human ORM ELISA kit obtained from Abcam PLC., USA (ab108854; sensitivity: 0.1 μg/mL; intra-assay CV%: 5.3%; inter-assay CV%: 10.0%; measuring range: 12.5–100.0 ng/mL) according to the manufacture’s instruction under the dilution ratio 1:10,000. Serum levels of LAC, cortisol, and CRP were determined via Human LAC ELISA kit (H263-1–2; sensitivity: 0.1 ng/mL; intra-assay CV%: < 12%; inter-assay CV%: < 10.0%; measuring range: 40–10,000 ng/mL), Human Cortisol ELISA kit (H094; sensitivity: 0.1 ng/mL; intra-assay CV%: < 12%; inter-assay CV%: < 10.0%; measuring range: 5–2000 ng/mL), Human CRP ELISA kit (H126; sensitivity: 0.01 mg/L; intra-assay CV%: < 12%; inter-assay CV%: < 10.0%; measuring range: 0.5–150 mg/L) obtained from Shanghai Branch of Nanjing Jiancheng Bioengineering Institute (Shanghai, China) according to the manufacture’s instruction without dilution.

### Statistical analysis

Statistical analysis was performed using IBM SPSS Statistics version 21.0 (IBM Corp., Armonk, NY, USA). Within-group comparisons were performed using the paired *t*-test or Wilcoxon signed rank test, and between-group differences were compared using one-way analysis of variance or Kruskal–Wallis H test for normality and non-normality data, respectively. The relationship between serum ORM and cortisol, lactate and CRP, and BCT RPE scores and VJH were analyzed by Pearson or Spearman correlation analysis according to the normality. The diagnostic value of ORM was evaluated by receiver operating characteristic (ROC) curve as the area under the ROC curve (AUC), sensitivity and specificity. The optimal cut-off values were obtained via Youden index approach [42]. Further, logistic regression was conducted to calculate the odds ratio (OR) of ORM cut-off value in predicting EIF. A *p* value < 0.05 was considered statistically significant.

## Results

### Characteristics of participants

Demographic characteristics and BCT RPE score are summarized in Table 1. According to the RPE score after BCT, a total of 12, 24 and 17 participants were classified into slight, moderate, and severe fatigue group (groups 1–3), respectively. The degree of EIF measured by RPE after BCT had significant difference among groups (post-BCT RPE score for groups 1–3: 11.33 ± 0.78, 13.33 ± 0.48, 16.00 ± 1.54, respectively, ranged from 10 to 20; *p* < 0.001). No significant difference was observed among groups in terms of demographic characteristics, age, height, weight, and body mass index (all *p* > 0.05).

**Table 1:** Demographic characteristics and BCT RPE score among groups (*N* = 53)

Item	Group 1 (*n* = 12)	Group 2 (*n* = 24)	Group 3 (*n* = 17)	*p* value
Age (year)	20.08 ± 1.24	20.00 ± 1.45	20.65 ± 1.62	0.398
Height (cm)	175.13 ± 2.71	175.54 ± 4.83	174.12 ± 5.70	0.572
Weight (kg)	68.14 ± 11.25	69.96 ± 9.09	67.58 ± 9.22	0.685
Body mass index	22.21 ± 3.54	22.69 ± 2.74	22.28 ± 2.72	0.771
Pre-BCT RPE score	8.83 ± 1.59	8.96 ± 1.30	9.47 ± 1.28	0.217
Post-BCT RPE score	11.33 ± 0.78	13.33 ± 0.48	16.00 ± 1.54	< 0.001

Data were shown as mean ± standard deviation. Between-group *p* value was calculated by Kruskal–Wallis H test

*BCT* basic combat training, *RPE* score of the Borg Rating of Perceived Exertion scale

### Change of exercise capacity (VJH) among groups

**Fig. 2:**
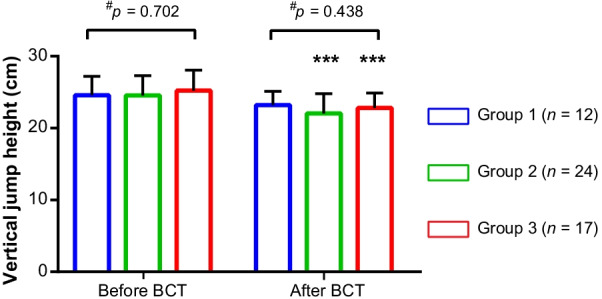
Vertical jump height among groups. Data are present as mean ± standard error of mean. ^#^Between-group *p* value, calculated by Kruskal–Wallis H test. ***Within-group *p* value < 0.001, compared with baseline (before BCT), calculated by Wilcoxon test. BCT, basic combat training

As shown in Fig. 2, before BCT, the VJH among the three groups were comparable (between-group *p* = 0.702). After 12-week BCT, the VJH of group 1 showed a decreasing trend from 24.58 ± 2.64 cm to 23.21 ± 1.92 cm (within-group *p* = 0.184), and that of group 2 and group 3 decreased significantly (both within-group *p* < 0.001), verifying the existence of EIF among groups: for group 1, the EIF did be very light as only slight perception of exertion existed without substantial decline of exercise capacity; but for groups 2 and 3, the degree of EIF increased as the average RPE score be higher with substantial decline of exercise capacity. The correlation analysis showed no correlation relationship between BCT RPE scores and VJH before and after BCT, and between changed BCT RPE scores and changed VJH during BCT (Additional file 1: Table S1), further implying the alternation of VJH and BCT RPE during the process of EIF may be not in a linear fashion.

### Level of blood ORM, LAC, CRP, and cortisol among groups

**Fig. 3:**
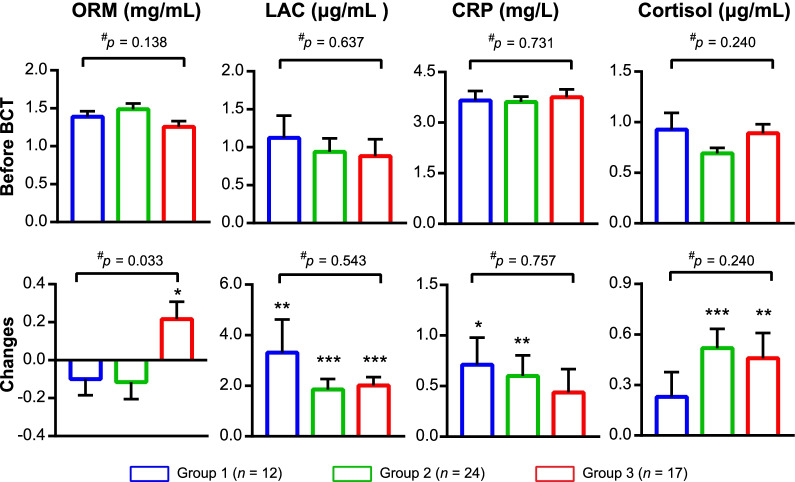
Level of ORM, LAC, CRP, and cortisol among groups. Data were shown as mean ± standard error of mean. Changes were calculated as the level after BCT minus that before BCT. ^#^ Between-group *P* value, calculated by Kruskal–Wallis H test. * Within-group *p* < 0.05, **p < 0.01, ***p < 0.001, levels of parameters after BCT compared with those before BCT, calculated by Wilcoxon test. BCT, basic combat training; CRP, C-reactive protein; LAC, lactate; ORM, orosomucoid

The levels of ORM, LAC, CRP, and cortisol are present in Fig. 3.

**Fig. 4:**
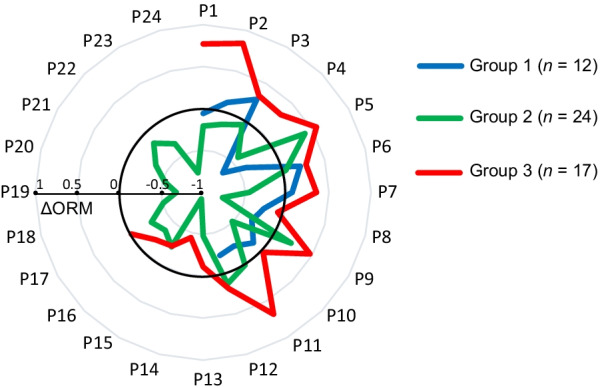
The change of orosomucoid (ΔORM) of each recruit in three groups. Pi (i = 1,2,3….24): the serial number of participants in specific group. ΔORM was calculated as the level after basic combat training minus that before basic combat training. The unit of ΔORM is mg/mL

Before BCT, the baseline level of ORM, LAC, CRP, and cortisol were comparable among groups (Fig. 3, all *p* > 0.05). After BCT, ORM had a significant increase in group 3 (0.22 ± 0.37 mg/mL, *p* = 0.039), and declined without significance in groups 1 and 2. The changes of ORM levels were significant among groups (Fig. 3, *p* = 0.033, individual data are shown in Fig. 4), indicating that serum ORM would specifically increase among population with severe EIF triggered by BCT.

The level of LAC, CRP, and cortisol almost increased among all groups after BCT, with all within-group *p* < 0.05 except the increase of CRP in group 3 and cortisol in group 1. However, the changes of these 3 indicators had no significant between-group difference (Fig. 3, *p* = 0.543, 0.757 and 0.745, respectively), implying that although BCT could increase their levels, the 3 indicators cannot distinguish the different degrees of EIF triggered by BCT.

The correlation analysis showed that the levels of serum ORM, LAC, CRP, and cortisol had some correlation relationships, but the correlation relationships varied among the before and after BCT (Additional file 1: Table S1), further illustrating the different alternation mode of these four parameters in indicating the degree of EIF.

### The potential diagnostic value of serum ORM in EIF

Since we found that after BCT, the changed level of ORM (ΔORM, calculated as the level after BCT minus that before BCT) was specifically increased significantly in severe EIF population (group 3, Fig. 3) but not in slight and moderate EIF population (groups 1 and 2), we further analyzed ROC curve analysis to evaluate its potential diagnostic value in severe EIF after BCT (post-BCT RPE score > 14).

**Fig. 5:**
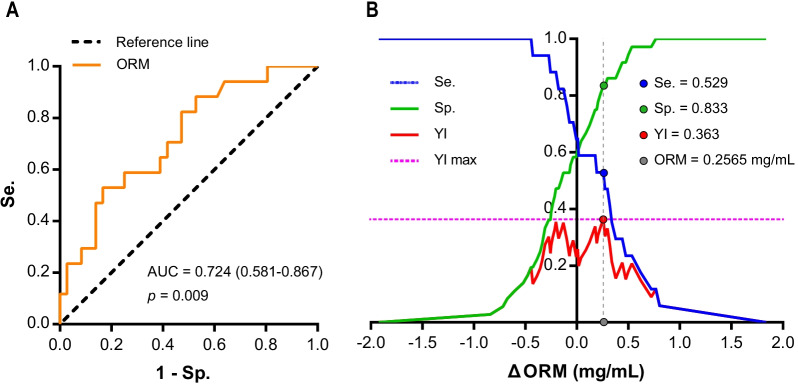
Diagnostic ability and cut-off value of ΔORM. **A** Receiver operating curve of ΔORM. **B** Sensitivity (Se.), specificity (Sp.) and Youden index (YI) at various cut-off points of ORM. AUC, area under the receiver operating characteristic curve; ΔORM, changed level of orosomucoid during basic combat training (BCT), calculated as the level after BCT minus that before BCT; YI max, the value of *y*-axis equals the maximal value of Youden index

The estimated AUC for ΔORM was 0.724 (95% CI 0.581–0.867, *p* = 0.009, Fig. 5A). As the maximal Youden index suggested, the recommended optimal cut-off value of ΔORM was 0.2565 mg/mL with sensitivity and specificity as 0.529 and 0.833 (Fig. 5B), and accuracy as 71.1% (38/53, Table 2).

**Table 2:** OR for severe EIF among recruits with ΔORM ≥ cut-off

ΔORM	Cases	Post-BCT RPE score		Incidence of severe EIF (%)	OR (95% CI)	*p* value of OR
		≤ 14	> 14			
< Cut-off value	38	30	8	21.1	Referent	0.009
≥ Cut-off value	15	6	9	60.0	5.625 (1.542–20.523)	

Cut-off value of ΔORM = 0.2565 mg/mL. Severe EIF was defined as post-BCT RPE score > 14

*BCT* basic combat training, *CI* confidence interval, *EIF* exercise-induced fatigue, *ΔORM* changed level of ORM during BCT, calculated as the level after BCT minus that before BCT, *OR* odds ratio

Further, logistic analysis of this dataset showed that after BCT, for recruits with ΔORM ≥ 0.2565 mg/mL, the odds for suffering from severe EIF were 5.625 times as large than the odds for those with ΔORM < 0.2565 mg/mL (95% CI 1.542–20.523, *p* = 0.009, Table 2).

In contrast, in order to verify ORM is more useful than LAC, CRP, and cortisol in diagnosing severe EIF population, we also calculated estimated AUC for changed level of LAC, CRP and cortisol. As shown in Table 3, the estimated AUCs for these three biomarkers failed to have significant diagnostic ability (*p* value of AUC > 0.05 for all).

**Table 3:** Estimated AUC area for ΔORM, ΔLAC, ΔCRP and Δcortisol

Item	AUC	Standard error of AUC	95% CI of AUC	*p* value of AUC
ΔORM	0.724	0.073	0.581–0.867	0.009
ΔLAC	0.587	0.085	0.421–0.753	0.313
ΔCRP	0.451	0.085	0.285–0.617	0.568
Δcortisol	0.493	0.091	0.315–0.672	0.939

Δ: changed level during basic combat training (BCT), calculated as the level after BCT minus that before BCT. *AUC* area under the receiver operating characteristic curve, *CI* confidential interval, *CRP* C-reactive protein, *LAC* lactate, *ORM* orosomucoid

All these analyses of potential diagnostic indicated that ΔORM may be an effective biomarker for the severe EIF triggered by BCT.

## Discussion

Fatigue is one of our body’s normal physical reactions to training. The degree of fatigue is actually a state which covers the interaction of both physical fatigue manifesting as the descending physiological function of the body to maintain a predetermined exercise intensity [1] and psychological fatigue manifesting as the subjective perception such as feeling tired and exhausted [43]. Although the Borg’s Rating of Perceived Exertion Scale innovatively established a correlation between physical and psychological state of EIF, making the degree of EIF measurable, it is still a kind of subjective evaluation in essence. Therefore, researchers have exerted efforts on exploring objective biomarkers for some specific population like athletes of swimming [44], marathon [45], soccer [46], etc. Military training, including basic combat training, is a central section to enhance combat effectiveness. Since EIF is inevitable during the process, precise diagnosis of EIF has important significance in adjusting training schedule, promoting training adaption, and preventing military-training injury.

Before our study, no confirmed biomarker was available for assessing EIF triggered by BCT. In our study, we observed the level of ORM together with LAC, cortisol and CRP, the potential indicators of EIF suggested by previous studies, during a 12-week basic combat training.

Our study illustrated that the level of LAC, cortisol and CRP increased among all groups after BCT, however, no significant difference was observed among groups, implying that although the three parameters may indicate the presence of training load, they could not distinguish EIF at different degree, thus cannot be biomarkers of EIF triggered by BCT. Previous studies also observed LAC, cortisol and CRP increased after exercise, which is consistent with our experimental results. Some studies regarded LAC as a “fatigue agent” and “signal molecule” in muscle fatigue [47], since acidosis strongly contributes to fatigue and lower work capacity has strong connection with lower pH [20]. However, other factors such as depleted glycogen levels [47] or a high carbohydrate diet [48] prior exercise can also influence blood LAC concentration. During BCT, though nutritive diet was supplied uniformly for each recruit, we did not explore the quantity of glycogen and carbohydrate, which may explain why LAC lacks the capacity to be detected as a biomarker. The increase of cortisol after BCT may be resulted from the stress response of exercise, which promoted the release of cortisol through feedback regulation [48]. But according to our results, recruits may have similar stress response to BCT, which had no correlation with the degree of EIF. Increase of serum CRP was also observed among civil servant with occupational burnout in British [49], and cycling athletes [50]. In this study, CRP was included as an analogue of ORM. Its failure in distinguishing EIF may underline the individualization and specificity of various acute-phase proteins, and the potential difference of physical mechanism in different exercises.

Although the LAC, cortisol and CRP failed to be a biomarker of EIF triggered by BCT, we discovered that the level of serum ORM specifically increased significantly among participants with severe EIF (post-BCT RPE score > 14), indicating ΔORM (changed level of ORM during BCT, calculated as the level after BCT minus that before BCT) to be a promising biomarker of severe EIF. Further analysis of ΔORM on its diagnostic value demonstrated that it had good discriminatory potential to diagnose severe EIF with estimated AUC as 0.724 (generally an AUC of 0.5 suggests no discrimination, 0.7 to 0.8 is considered good, 0.8 to 0.9 excellent and > 0.9 outstanding [51, 52]). The optimal cut-off value of ΔORM is 0.2565 mg/mL as recommended by the maximal Youden index. Although the value seems small, the logistic analysis based on this dataset showed that after BCT, recruits with ΔORM ≥ 0.2565 mg/mL had higher odds for suffering from severe EIF, as 5.625 times as large than those with ΔORM < 0.2565 mg/mL, which also revealed its potency as a promising biomarker. Previous studies reported that ORM could interact with skeletal muscle and inhibit protein breakdown [53], and increase muscle bioenergetics and sustain physiological endurance via upregulating the ATP production through AMPK signalling pathway [17, 54]. When recruits felt exhausted (severe EIF during BCT), the energy supply was likely to be under compensation stage, therefore may triggered the increase of ORM. Oppositely, when the recruits could continue exercise easily, the compensation stage of energy may not be motivated, which could explain why ΔORM could indicate severe EIF (RPE score > 14) while not slight and moderate EIF (RPE score ≤ 14). But, anyway, the exact mechanism is needed to be verified by studies in the future.

This study initially explored the association of ORM and EIF triggered by BCT among male recruits undergoing Chinese BCT, revealed ΔORM to be a promising biomarker of severe EIF, and preliminarily estimated its optimal cut-off value as 0.2565 mg/mL, therefore, it can serve as a reference for EIF assessment during BCT.

Our study also has some limitations. Firstly, this study is only an observational study of 53 recruits. All participants were selected by their willingness without randomization. No specific sample size calculation was performed. Therefore, the diagnostic value of ΔORM should be verified and tested by larger diagnostic studies. Secondly, the exercise was confined to BCT, whether ΔORM could indicate EIF triggered by other types of exercise remains unknown. Thirdly, we only investigated male recruits in this study, therefore, our conclusions may not be applicable to female recruits. Fourthly, since the researchers were forbidden to collect blood sample more than twice, the timeline of ORM increase during BCT was not explored. In addition, exercise-induced fatigue may involve many such as physical, psychological, cognitive, and motor appropriate etc., biomarker or ORM may only be a part of them. More studies about holistic integrate function of human, and to establish a comprehensive evaluation framework of EIF may add to the accuracy of diagnosis.

## Conclusion

In summary, we found that ORM increased specifically among recruits with severe EIF after BCT, revealed the diagnostic value of ΔORM in discriminating severe EIF from slight and moderate EIF, and estimated its potential optimal cut-off value as 0.2565 mg/mL. Thus, ORM might be a novel and promising biomarker for the assessment of EIF triggered by BCT. Whether the diagnostic performance of serum ORM together with classical parameters of EIF will be better than serum ORM alone is valuable to be explored in the future.

## Supplementary Information

**Additional file 1: Table S1.** The correlation between RPE, VJH, serum ORM, serum CRP, serum C, and serum LAC.

## Data Availability

The datasets used and/or analysed during the current study are available from the corresponding author on reasonable request.

## Abbreviations

BCT: Basic combat training
CRP: C-reactive protein
EIF: Exercise-induced fatigued
LAC: Lactate
ORM: Orosomucoid
ΔORM: Changes of orosomucoid
ROC: Receiver operating characteristic
RPE: The degree of exercise-induced fatigue measured by Borg’s Rating of Perceived Exertion Scale (Borg-RPE-Scale®), i.e., RPE score
