# Supplementary material for: Orosomucoid: a promising biomarker for the assessment of exercise-induced fatigue triggered by basic combat training
Source: BMC Sports Sci Med Rehabil. 2022 Jun 3;14:100. doi: 10.1186/s13102-022-00490-6 (PMC9166395; doi:10.1186/s13102-022-00490-6)
Supplement: Supplementary file 1 — Additional file 1: Table S1. The correlation between RPE, VJH, serum ORM, serum CRP, serum C, and serum LAC. [file 13102_2022_490_MOESM1_ESM.docx]

**Supplementary File**

**Table S1.** The correlation between RPE, VJH, serum ORM, serum CRP, serum C, and serum LAC.

| Variables | Correlation coefficient | | | | | | | | | | | | | | | | | |
| --- | --- | --- | --- | --- | --- | --- | --- | --- | --- | --- | --- | --- | --- | --- | --- | --- | --- | --- |
|  | RPE1 | RPE2 | ΔRPE | ORM1 | ORM2 | ΔORM | CRP1 | CRP2 | ΔCRP | C1 | C2 | ΔC | LAC1 | LAC2 | ΔLAC | VJH1 | VJH2 | ΔVJH |
| RPE1 | / |  |  |  |  |  |  |  |  |  |  |  |  |  |  |  |  |  |
| RPE2 | 0.249 |  |  |  |  |  |  |  |  |  |  |  |  |  |  |  |  |  |
| ΔRPE | **-0.477^**^** | **0.662^**^** |  |  |  |  |  |  |  |  |  |  |  |  |  |  |  |  |
| ORM1 | 0.002 | -0.165 | -0.191 |  |  |  |  |  |  |  |  |  |  |  |  |  |  |  |
| ORM2 | 0.070 | **0.334^*^** | 0.211 | -0.144 |  |  |  |  |  |  |  |  |  |  |  |  |  |  |
| ΔORM | 0.038 | **0.275^*^** | 0.234 | **-0.889^**^** | **0.535^**^** |  |  |  |  |  |  |  |  |  |  |  |  |  |
| CRP1 | -0.039 | 0.068 | 0.125 | -0.186 | -0.072 | 0.123 |  |  |  |  |  |  |  |  |  |  |  |  |
| CRP2 | -0.186 | -0.091 | 0.125 | **-0.526^**^** | -0.093 | **0.390^**^** | **0.301^*^** |  |  |  |  |  |  |  |  |  |  |  |
| ΔCRP | -0.179 | -0.101 | 0.055 | -0.214 | 0.018 | 0.178 | **-0.664^**^** | **0.474^**^** |  |  |  |  |  |  |  |  |  |  |
| C1 | -0.063 | 0.125 | 0.103 | **-0.659^**^** | 0.011 | **0.546^**^** | **0.409^**^** | **0.341^*^** | -0.111 |  |  |  |  |  |  |  |  |  |
| C2 | -0.020 | 0.057 | 0.060 | **-0.645^**^** | 0.139 | **0.617^**^** | **0.280^*^** | **0.524^**^** | 0.123 | **0.501^**^** |  |  |  |  |  |  |  |  |
| ΔC | -0.011 | -0.036 | 0.002 | -0.229 | 0.092 | 0.249 | -0.012 | **0.381^**^** | **0.273^*^** | -0.167 | **0.730^**^** |  |  |  |  |  |  |  |
| LAC1 | -0.064 | -0.125 | -0.068 | **0.554^**^** | -0.084 | **-0.529^**^** | 0.128 | **-0.302^*^** | **-0.305^*^** | **-0.305^*^** | **-0.476^**^** | -0.260 |  |  |  |  |  |  |
| LAC2 | 0.055 | -0.136 | -0.209 | 0.087 | 0.139 | -0.042 | -0.200 | -0.069 | 0.172 | -0.112 | -0.161 | -0.042 | 0.165 |  |  |  |  |  |
| ΔLAC | 0.122 | 0.023 | -0.106 | **-0.378^**^** | 0.172 | **0.381^**^** | -0.159 | 0.206 | **0.315^*^** | 0.227 | 0.270 | 0.160 | **-0.464^**^** | **0.715^**^** |  |  |  |  |
| VJH1 | -0.136 | 0.106 | 0.130 | -0.186 | 0.187 | 0.214 | -0.026 | -0.093 | -0.095 | 0.135 | 0.203 | 0.128 | -0.041 | -0.130 | -0.121 |  |  |  |
| VJH2 | 0.052 | -0.055 | 0.014 | -0.130 | 0.046 | 0.086 | 0.075 | -0.092 | -0.183 | 0.106 | 0.021 | -0.094 | 0.068 | -0.146 | -0.119 | **0.505^**^** |  |  |
| ΔVJH | 0.210 | -0.111 | -0.137 | 0.095 | -0.161 | -0.185 | 0.060 | -0.070 | -0.099 | 0.034 | -0.157 | -0.216 | 0.059 | 0.066 | 0.097 | **-0.600^**^** | **0.320^*^** | / |

1: level of variables before basic combat training (BCT); 2: level of variables after BCT; Δ: changed level of variables, calculated as the level after BCT minus that before BCT. RPE: the degree of exercise-induced fatigue measured by Borg’s Rating of Perceived Exertion Scale (Borg-RPE-Scale®), i.e., RPE score; ORM: serum orosomucoid; CRP: serum C-reactive protein; C: serum cortisol; LAC: serum lactate; VJH: vertical jump height. * The *p* value of correlation coefficient < 0.05; ** The *p* value of correlation coefficient < 0.01. The correlation coefficient was calculated via Pearson correlation analysis.
